# COVID-19 Can Catalyze the Modernization of Medical Education

**DOI:** 10.2196/19725

**Published:** 2020-06-12

**Authors:** Cathy Hsi Chen, Alexander Joseph Mullen

**Affiliations:** 1 Department of Medicine University of Mississippi Medical Center Jackson, MS United States

**Keywords:** medical education, health professions education, medical school, curriculum design, flipped classroom, preclinical education, COVID-19, coronavirus, medical student

## Abstract

Amid the coronavirus disease (COVID-19) crisis, we have witnessed true physicianship as our frontline doctors apply clinical problem-solving to an illness without a textbook algorithm. Yet, for over a century, medical education in the United States has plowed ahead with a system that prioritizes content delivery over problem-solving. As resident trainees, we are acutely aware that memorizing content is not enough. We need a preclinical system designed to steer early learners from “know” to “know how.” Education leaders have long advocated for such changes to the medical school structure. For what may be the first time, we have a real chance to effect change. In response to the COVID-19 pandemic, medical educators have scrambled to conform curricula to social distancing mandates. The resulting online infrastructures are a rare chance for risk-averse medical institutions to modernize how we train our future physicians—starting by eliminating the traditional classroom lecture. Institutions should capitalize on new digital infrastructures and curricular flexibility to facilitate the eventual rollout of flipped classrooms—a system designed to cultivate not only knowledge acquisition but problem-solving skills and creativity. These skills are more vital than ever for modern physicians.

Amid the coronavirus disease (COVID-19) crisis, we have witnessed true physicianship as our frontline doctors apply clinical problem-solving to an illness without a textbook algorithm. Yet, for over a century, medical education in the United States has plowed ahead with a system that prioritizes content delivery over problem-solving and passive learning over active learning. Trainees develop problem-solving skills despite our preclinical education system, not because of it. A smattering of institutions has begun to reinvent, but for what may be the first time, we have a chance to push through necessary change on a broader scale. In response to the pandemic, medical educators have scrambled to conform curricula to social distancing mandates. The resulting online infrastructures could enable us to achieve what education leaders have long advocated [1]—eliminate the traditional classroom lecture in favor of active learning.

According to the Association of American Medical Colleges, lectures comprise half of medical school teaching, with 86 percent occurring in the first 2 years [2]. Despite their ubiquity, in-person lectures are increasingly rejected by students. In 2019, 49% of preclinical students reported “never” or “only occasionally” attending lectures, up from 41% 2 years prior [3]. Instead, they are turning to online material.

And why not? Online lecture videos allow students to peruse content at their own pace, as well as pause, review, and adjust playback speed. Research supports what students implicitly understand: online lectures are noninferior for learning, and often actually better [4].

For disheartened faculty teaching to half-filled auditoriums, the instinct may be to incentivize attendance. But it is time to teach the way modern physicians learn rather than how traditional educators teach. Class time should be used for active learning and learner-educator interaction—not content delivery. Many students feel their lecture-based, preclinical education poorly prepares them for clinical rotations [5]. As resident trainees, we are acutely aware that knowing content is not enough. The path from “know” to “know how” can be treacherous.

This is where educators can provide value beyond lecturing—by engaging learners, guiding their clinical problem-solving, integrating preclinical material into the clinical context, and providing corrective feedback. Traditional lectures are inherently unable to catch and address these individual reasoning deficits in real time. When we commit class time to lectures, we waste faculty expertise and eschew evidence-based learning.

In “flipped classrooms,” students consume content online before working with educators to apply knowledge in group sessions (eg, through problem-based learning). Compared to traditional medical lectures, flipped classrooms produce better learning outcomes [6], especially in higher-order thinking [7]. Perks include greater class attendance [7] and teacher-student satisfaction [8]. Meta-analyses are limited because implementations vary, but research suggests that as the methodology matures, outcomes will further improve [7].

As problem- and case-based learning sessions become more prevalent, we will undoubtedly witness them evolve in their effectiveness, with space to explore other educational approaches (eg, patient simulators, augmented reality) as well. In addition, with the right analytics, flipped classrooms can exploit big data in ways traditional curricula cannot. Educators can track progress and target active learning sessions to actual knowledge gaps identified by frequent no-stakes tests.

Instructor time and cost-effectiveness are perhaps the leading critiques of flipped classrooms. The pandemic, however, is an inescapable impetus to transition content online. With infrastructure in place and costs already sunk, the barriers to enacting flipped classrooms (once social distancing guidelines relax) will be the lowest they have ever been. A key remaining concern—that more faculty are needed to coordinate group sessions—may be addressed in several ways. With content online, faculty can afford to meet with students less frequently. They might also enlist more teaching assistants: upper-level medical students, residents, fellows, and clinical faculty, all of whom spend disappointingly little time interacting with early learners.

Flipped classrooms are not new, and medical schools have been moving in their direction, but slowly. Change is difficult in storied institutions. In the setting of a crisis, however, change is the new normal. The Liaison Committee on Medical Education, the accrediting body for US and Canadian Medical Schools, has acknowledged that broad changes to the mechanisms of learning need to occur [9]. As such, they are granting institutions significant curricular flexibility, which can be harnessed to implement novel pedagogy.

In 1913, Dr William Osler said, “The lecture has its value, but its day has gone, and it should give place to other methods better adapted to modern conditions” [10]. We should not emerge from the pandemic only to revert to a preclinical education system even Dr Osler found outdated. We must make the most of our new digital infrastructures and curricular flexibility to facilitate the eventual rollout of flipped classrooms—a system deliberately designed to cultivate not only knowledge acquisition but problem-solving skills and creativity. These skills are more vital than ever for modern physicians.

## Abbreviations

COVID-19: coronavirus disease
